# Treatment management during the adolescent transition period of girls and young women with Mayer-Rokitansky-Küster-Hauser syndrome (MRKHS): a systematic literature review

**DOI:** 10.1186/s13023-016-0536-6

**Published:** 2016-11-16

**Authors:** Anke Wagner, Sara Yvonne Brucker, Esther Ueding, Dagmar Gröber-Grätz, Elisabeth Simoes, Katharina Rall, Andrea Kronenthaler, Norbert Schäffeler, Monika A. Rieger

**Affiliations:** 1 Institute of Occupational and Social Medicine and Health Services Research, University Hospital Tuebingen, Wilhelmstraße 27, 72074 Tübingen, Germany; 2 Centre of Women’s Health, University Hospital Tübingen, Calwerstraße 7, 72076 Tübingen, Germany; 3 Women’s Health Research Institute, Calwerstraße 7, 72076 Tübingen, Germany; 4 Institute of General Practice, University of Tübingen, Österbergstraße 9, 72074 Tübingen, Germany; 5 Department of Psychosomatic Medicine and Psychotherapy, University Hospital Tübingen, Germany, Osianderstraße 5, 72076 Tübingen, Germany

**Keywords:** Transition, Transition care, Rare disease, Systematic literature review

## Abstract

**Introduction:**

In health services research, there is a special emphasis on the transition from adolescence into adulthood. During this transition period, adolescents change from pediatric to adult medical care. This process must be carefully structured, particularly when special medical care is required. Challenges and difficulties become apparent particularly in the case of rare diseases. This is increasingly so when the rare disease affects the adolescence-specific development of patients, such as Mayer-Rokitansky-Küster-Hauser syndrome (MRKHS), also known as Müllerian agenesis.

**Methods:**

A systematic literature review identified the care requirements of girls and young women with MRKHS, as well as studies of medical care during the adolescent transition period for various other diseases. This investigation was carried out in the years 2012 and 2013, and was updated in 2014/2015. In addition, the reference lists of the identified studies were reviewed.

**Results:**

Nine publications on MRKHS and ten publications on the transition from adolescence to adulthood were included. Medical care requirements and measures were identified for the following areas: diagnosis during adolescence and organization of medical care, reactions to the diagnosis, functional infertility, psychological stress and threat to self-image, contact with others, and dealing with MRKHS coping strategies.

**Discussion:**

There is still a great demand for research in the area of care during the transition period from adolescence into adulthood, particularly for rare diseases. The recommendations for treating MRKHS patients derived from the literature should be implemented and evaluated with regard to their effectiveness.

## Background

The transition from adolescence to adulthood – particularly for adolescents with special medical needs – includes special requirements for treatment management regarding disease-specific and phase-specific aspects [1]. “Transition is a multi-dimensional and multidisciplinary process, which not only addresses the medical needs of the adolescents when transitioning from pediatric to adult care, but also includes psychosocial, scholastic, and professional aspects” [1]. The term “transfer”, on the other hand, describes a single aspect of the considerably longer transition process – and here it means the direct transfer from pediatric to adult medical care [1]. Both terms are essential for understanding transition care. The German Advisory Council on the Assessment of Developments in the Healthcare System describes the current state of transition care in Germany [1]:

- Due to a lack of coordination of the necessary processes, patients run the risk of receiving insufficient treatment during the transfer to adult care. This insuffient care can lead adolecents to discontinue therapy or to stop taking prescribed medications.
- This lack of coordination means that transfers into adult care are unplanned. There is no room for coordinated support in the transition process.
- During adolescence, young patients not only have to deal with a chronic disease, but also deal with compelling questions regarding their sexuality, leaving their childhood homes, and planning their professional lives.

In Germany, initial steps are currently being taken to improve transition management – see Berliner TransitionsProgramm (BTP) (“Berlin Transition Program”) [2] and the transition module “Erwachsen werden mit ModuS: Fit für den Wechsel (“Coming of Age with ModuS: Fit for the Change”) [3] – wherein data on the effectiveness of these approaches must still be evaluated.

Previous approaches to transition management do not put any special emphasis on rare diseases, although difficulties in treatment management could become particularly apparent in these cases. A disease is categorized as “rare” when less than five to ten persons per 10,000 are afflicted. One disease that represents a particular challenge with regard to transition management is Mayer-Rokitansky-Küster-Hauser syndrome (MRKHS). The prevalence of MRKHS is given as 1 in 5000 female births [4]. This is characterized by the congenital absence of the uterus and the upper 2/3 the vagina with a normal 46,XX karyotype and a normal, age-appropriate hormone profile, since functional ovaries are present [4]. Therefore, the development of secondary sexual characteristics occurs normally [4, 5] and thus the diagnosis MRKHS is generally made late, at the beginning of puberty due to the lack of onset of menstruation (primary amenorhhea) or, seldom, due to the impossibility of sexual intercourse [4]. On physical examination, patients have normal breast development, normal secondary sexual body proportions, body hair, and hymenal tissue [5], which is one of the reasons for misdiagnosis up to 40 % at the first contact to a physician [6]. On the other hand, this means, that these young women get their diagnosis of “being not a complete women” in the sensible phase of puberty. The presence of functioning ovaries means patients are basically fertile; i.e. surrogacy is possible; but the lack of a uterus means pregnancy cannot be carried out by the patients themselves [4]. As the vagina is either not developed or extremely short, vaginal intercourse is seldom possible for women who have not received treatment [4]. In approximately one third to one half of all cases, patients have concomitant congenital malformations, especially of the urinary tract and skeleton [4, 7]. Therefore, evaluation for associated congenital, renal, or other anomalies is also essential [8].

After the diagnosis, the patient should be offered counseling to a specialiced center, which offers also psychological counseling to emphasize that a normal sex life will be possible after a neovagina has been created, especially as long-term results are very good [9] but also to tell them how to deal with the problem of infertility, wich comes up normally later, at the age of mid to late twenties.

One of the goals to strengthen their self-empowerment towards being a nomal woman is the creation of a neovagina, which looks like a normal one and where no use of lubricans is needed.

Therefore, in our opinion, there are only two first line treatment options

- The nonsurgical treatment: this means that patients are asked to manually place successive dilators on the perineal dimple for 30 min to 2 h per day. Another option is sitting on a bicycle seat stool. The bicycle seat stool provides the perineal pressure. Use of dilators in the management of MRKHS is appropriate and successful for mature, highly motivated patients who wish to avoid surgery because the treatment endures several months [5] and is very painful and dilation of the uretra could occure, It is no option, if the vaginal dimple is to short.
- The surgical treatment should avoid big scars and transplanted tissue like skin, bowl or peritoneum. Therefore the laparoscopic-assisted creation of a neovagina is the treatment of choice. This method creates after a few months a neovagina by minimal invasive access with a normal epithelium after full epithelialization [10, 11].

Apart from these treatment options infertility may be a more difficult aspect for the patients to accept [5].

## Purpose and methods

The past research focused mainly on physical aspects and on evaluation of surgical methods. Other treatment requirements of patients with MRKHS focusing on the transition period are due to our knowledge discussed less frequently. This systematic literature review aims to determine treatment requirements of girls and young women with MRKHS during transition and to develop recommendations for the transition management of this target group. The derived recommendations were developed in the context of health services research in Germany.

**Fig. 1:**
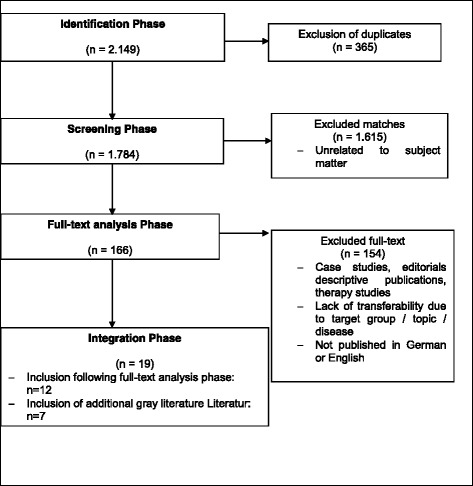
Study Extraction

The literature review was done within the framework of the research project “TransCareO” [12, 13]. The systematic literature search was done using the PubMed database from June 2012 to March 2013 (updated 2014/2015). Detailed information on the search strategy and selection process for inclusion in the study is described in Fig. 1 and Table 1.

**Table 1:** Additional information about the inclusion process

Additional information about the inclusion process during the literature research
• Identification Phase To identify appropriate studies, a systematic literature search was carried out using the PubMed database (online portal of the National Library of Medicine) from June 2012 through March 2013. The literature search was updated in 2014/2015 to include newer publications. The search was carried out for clinical picture, target group, treatment management, and outcome using keywords and keyword combinations defined by the project team. The search was performed with a broad scope, also including findings from publications on transition management for other relevant diseases when deriving recommendations. This was necessary, since few studies on transition for rare diseases could be found in 2012 and 2013. Two search strategies were applied: - Search strategy 1 comprised a search for publications generally related to the transition phase. The following keywords were used: **“**young girl AND puberty AND disease course“ and **“**girl AND transition“ - Search strategy 2 comprised searching for publications dealing with the disease MRKHS in the context of transition. The following catchwords were used: **“**parents AND MRKH“, “female AND adolescent AND gential malformation AND MRKH”, “MRKH AND transition“, **“**rokitans* AND organisation“, “female AND adolscent AND gential malformation AND transition“, “female AND rokitans* AND transfer”, “female AND adolscent (Mesh Terms) AND rokitans* AND disease management AND quality of life” The search in PubMed using both search strategies resulted in a total of 2,149 hits.
• Screening Phase After excluding duplicates (n=365), all publications found in the search were first evaluated according to title and abstract by two reviewers from the project team, independently from one another, with regard to suitability for the study question. This resulted in the exclusion of 1,615 hits. After a positive evaluation by both reviewers, the full-text was used for further analysis.
• Full-text Analysis Phase Following the screening phase, 166 hits were available for the full-text analysis. The full texts were read by a third person who made the final decision on inclusion in this review, and extracted the necessary content from the publications. The reference lists of the full-text publications read were also viewed for possible other suitable publications. In the course of this process, an additional 154 hits were excluded (editorials, descriptive publications, and therapy studies). Publications were excluded that were not written in either German or in English. Studies were also excluded if, due to the target group, the subject, or the disease, there was no transferability to the situation of patients with MRKHS.
• Integration Phase A total of 12 publications were included from the full-text analysis and an additional 7 publications from the reference lists of the included publications were integrated into the literature review (4, 7, 14-30).

## Results

A total of twelve studies [4, 7, 14–23] and seven theoretical works [24–30] were included in the literature review (Tables 2 and 3).

**Table 2:** Studies on MRKHS

Authors	Study design	Objective	Country	Numbers (Participants, Studies)	Instruments
Bean et al. (2009)	Narrative literature review	Examination of psychological effects and quality of life following the diagnosis and treatment of MRKHS	USA	31 Studies (differing methodologies)	none
Heller-Boersma et al. (2007)	Randomized Control Trial	Examination of the effectiveness of group therapy based on a cognitive behavior approach with regard to the psychological outcome before therapy, after therapy (7 weeks), and 3 months after therapy in women with MRKHS	UK	Intervention Group (*n* = 19) (response rate: baseline 100 %, after treatment 84 %, follow-up 84 %; age: 28.9, SD 10.0) Control Group (*n* = 20) (response rate: baseline 100 %, after treatment 80 %, Follow-up 80 %; Alter: 27.6, SD 7.1)	Symptom Checklist (SCL-90-R)/Rosenberg Self-Esteem Scale (RSE)/Impact of Events Scale (IES)/Short version of the Inventory of Interpersonal Problems (IIP-32)
Heller-Boersma et al. (2009)	Cross-sectional study	Examination of the psychological effect of MRKHS when comparing women with MRKHS and healthy women	UK	66 women (response rate: 20 %; age: 27.9, SD 1.0) 31 healthy women as a control group (response rate: 73.8 %; age: 27.8, SD 1.5)	Symptom Checklist (SCL-90-R)/Rosenberg Self-Esteem Scale (RSE)/Inventory of Interpersonal Problems (IIP-32)/Eating Disorder Inventory (EDI)
Holt et al. (2003)	Qualitative study	Examination of personal experiences of women with MRKHS, to gain insight into the psychological, social, and emotional consequences of diagnosis and treatment	UK	7 women (response rate: 17.5 %; age: 18–37)	Semi-structured interview guide
Huber et al. (2009)	Narrative literature review	Overview on the state of research on psychological aspects of women with MRKHS	Germany	43 studies (differing methodologies)	none
Loeser et al. (2002)	Qualitative study	Examination of mother-daughter relationships throughout the course of peri- and postoperative creation of a neovagina	Germany	8 women (response rate: no information; age: 14–21) 7 mothers (no additional information)	Structured interview guide
Patterson et al. (2014)	Qualitative study	Examination of the importance of MRKHS to gain insight into social challenges during the transition into adulthood	Scottland	5 women (response rate: no information; age: 18–22)	Semi-struktured interview guide
Weijenborg et al. (2000)	Cross-sectional study with pre-post design	Evaluation of the effectiveness of group therapy with regard to psychological burden before, at the beginning, and after treatment of women with MRKHS	Netherlands	17 women (for analysis: information from 15 women; age: 27.1; SD 10.0)	Symptom Checklist (SCL-90-R)

**Table 3:** Studies on Transition

Authors	Study Design	Main Topic	Country	Numbers (Participants, Studies)	Instruments	Main Results
Castrejón (2012)	Narrative literature review	Overview of information available in the literature on transition units with regard to rheumatic diseases	USA	46 studies (differing methodologies)	none	Recommendations for interacting with adolescent patients – Respect, confidentiality, avoiding mistakes, continuity, autonomous visits without parents, use of the HEADSS assessment Components of a transition unit – Early age, transition coordinator, transition program, involvement of pediatricians and physicians for adults, parents, multidisciplinarity , qualified personnel, evaluation
Crowley et al. (2011)	Systematic literature review	Review of the efficacy of transition programs in young patients between the ages of 11 and 25 with chronic diseases or disabilities and the identification of successful components	UK	10 studies (Evaluation of interventions during transition, but with different methodology)	none	Patients – Disease-related education (4x successful intervention) – Improved education and skills traning (2x successful intervention) Healthcare Facility – Transition coordinator (2x successful intervention) – Liaison between pediatric and adult hospital (3x successful intervention) Service offers – Separate clinics for young adults (3x successful intervention) – Telephone support service (1x successful intervention) – Appointment reminders by telephone (2x successful intervention)
Forbes et al. (2002)	Systematic literature review	Identification and evaluation of practices that assure adolecents with chronic diseases and disabilities of continuity during the transition	UK	Search A: 61 studies (Identification of Best-Practice.models) Search B: 39 studies (inclusion of five diseases) Search C: 26 studies (interview of key persons)	none	Structural component – Transition worker; transition teams; professional continuing education; information for specialists; use of existing services; inter- and intra-organization of networks and arrangements; organizational planing; theoretical framework; promotion of equality and accessibility. Process components – Preparation for the transfer; active management of the transition; case management; responsibility for the process; strengthening of therapeutic relationships; representation of interests; joint care management; flexibility with regard to the transfer; specific communication systems; regular assessment of provision of services. Result components – Disease-specific or general outcomes like satisfaction. Other outcome components are benchmarks of how service quality can be measured. Components of the practice regarding young people: – Specific services provided; developing competence in autonomy and self-determination; support for psychosocial development; inclusion of young people; peer Einbindung der junge Leute; involvement of peers; support for the changed relationship with parents/caregivers; making appropriate choices; availability of information; concentration on young people’s strengths for their future development Components in practice regarding parents and caregivers: – Support adjusting to the changed relationships to the young people; inclusion of parents in work scheduling; family-centered approach and provision of information.
Price et al. (2011)	Qualitative Study	Evaluation of a transition model based on interviews with young adults with diabetes mellitus	UK	11 young adults (age: 16–18 years) 2 young adults after 1 year	Semi-structure interview guide	Initial topics: – The transition process – Experience with, and organization of the transfer – Organization of services – Information and education – Health counselling – Integration. Primary topics – appropriate health care of young people – Recognizing individuality in health care

The studies were also evaluated with regard to their internal validity using Stein's unbiased risk estimate (SURE) criteria [31–33]. Care requirements for girls and young women with MRKHS were extracted from existing literature. Similarly, based on the care requirements identified, recommendations for treatment management were derived for this disease. In the following, recommendations will be presented for individual aspects of treatment management.

### Diagnosis during adolescence and organization of medical care

MRKHS is commonly diagnosed in early to late adolescence [24], when menarche fails to occur. For many young women, the first menstrual period symbolizes their female identity and maturity, and therefore contributes significantly to identity formation [24]. At the time of diagnosis, there is a risk that young women will perceive themselves as fundamentally different from their contemporaries [19, 24]. In addition, congnitive immaturity, lack of experience, and “black-and-white thinking” can lead to negative self-assessment and damaging coping strategies in dealing with the malformation [24]. Another problem at this sensitive age is dealing with a disease that requires discussing intimate topics, such as intercourse and sexuality, with other people [14]. One study reports that adolescents in particular lack knowledge about this disease and its significance [14]. In consequence, this can mean adolecents are incapable of effectively participating in the decision-making process regarding their own therapy, and therefore can lead to them developing the feeling that they have no choice or control over planned treatment methods [14]. In the publications analyzed, there is little information on how medical care and treatment was experienced. One study reports that women with MRKHS did not fully understand the medical terminology used by their caregivers [14]. These patients experienced the initial treatment period as a time of uncertainty [14]. Medical care was experienced as being limited to the physical examination, which mainly dealt with the physical aspects of the malformation [14]. In this case the patients criticized the lack of information and impersonality of provided care [14]. This led to patients feeling isolated and insufficiently supported in dealing with the disease and treatment [14]. Patterson et al. reported that mothers of patients became more protective of their daughters following diagnosis, took control, and were the principal communicators with medical staff, which is why patients did not see themselves as the main focus of treatment [19]. They also reported that medical personnel did not recognize or acknowledge the growing autonomy of the young adults during the course of treatment [19].

A recommendation for transition management can be derived from this: young women should be informed that, without treatment, there is little to no possibility of sexual intercourse [4]. The type of communication and the doctor-patient relationship are of particular importance in this context. Young patients should always be the focus of treatment and therapy, and should be allowed to play an active roll in the decision-making process [19]. It may also be desirable to conduct talks with young patients without their parents present, to enable intimate aspects such as sexuality to be discussed openly [19].

### Reactions to the diagnosis

Patients report numerous emotions in reaction to their diagnosis [24]. Positive reactions of relief as well as negative emotions were possible [24] – however, the negative expressions appear to predominate. Examples described in other studies include shock, confusion, fear, depression, suicidal thoughts, isolation, shame, guilt, and denial [9, 24]. One review mentions feelings of loss and fear [15]. In addition, as self-reported by some of the girls and young women, the impact of the diagnosis was so emotionally devastating that they were unable to fully absorb or understand the information about therapy and treatment given at that time [24].

It is therefore recommended that the emotional reactions of the patients to the diagnoses be allowed for with regard to further information about the disease and treatment possibilities (information management), and to repeat and explain important information at a later point in time [24].

### Functional infertility

The infertility that is a consequence of the disease is difficult for women with MRKHS to accept [15]. Huber et al. elaborate that this aspect of infertility, particularly over time, influences the further well-being of women with MRKHS [4].

It appears to be very important, therefore, to address this aspect comprehensively with the patients and to inform them of alternative options (e.g. adoption) [15].

### Psychological stress and threat to self-image

Three studies indicate that women with MRKHS tend to have increased psychological stress [7, 16, 17]. In their study, Heller-Boersma, Schmidt, and Edmonds [17] show that women with MRKHS have significantly higher values regarding risk to phobias and psycoticism than a healthy control group, and show similar tendencies for depression and fear. Furthermore, they report that in a direct comparison with a healthy control group, women with MRKHS have lower self-esteem [17]. MRKHS is also commonly experienced as a threat to one’s own identity and self-image [19, 24]. Due to the disease, role models considered distinctly female are challenged [19]. On one hand, the body is perceived as a threat [24]; a feeling that can increase when other malformations related to the disease are diagnosed [24]. On the other hand, social and sexual roles are questionned [14, 17, 19]. Women with MRKHS can also feel a loss of a sense of normality and belonging, in that they perceive themselves as being different than their peers [14]. This can lead to negative self-image developing in women with MRKHS, such as feeling different, defective or flawed, sexually inadequate, worthless, unloveable, or not in control [24]. There are also reports of feeling incomplete, for example when bearing children is considered as a fundamental female role [19].

During treatment, the affected women should be offered continuous guidance [24] and psychological support to cope with the diagnosis [19, 24]. Screening for individual psychological stress in girls and young women with MRKHS is also recommended [15].

### Contact with others

Contact with others is challenging for women with MRKHS. To some extent, the disease is experienced as a social tabu [19]. Patients frequently have the strong desire to keep the diagnosis secret [14, 19]. There are also predominant worries about other people’s reactions; both positive and negative reations were experienced [14, 19]. In addition, it is shown that in their contact with other people, it is sometimes impossible for women with MRKHS to participate in certain conversations with their peers [19]. There have also been occasional reports of difficulties dealing with children or pregnant women [19]. In particular, one study shows that women with MRKHS do not feel understood by their social environments – they state that only other women with MRKHS can understand their situation and experiences [19]. They express a strong desire to exchange feelings and experiences directly with other patients of the same age [19]. Another challenge is dealing with intimacy. Patients find it difficult to find the right time to inform their partners about their condition [19]. This is due, in part, to fears that the relationship will be ended because of the diagnosis and resulting effects on family planning [19]. Another study examined the course of MRKHS and individual mother-daughter relationships [18]. This study showed that patients with a healthy relationship with their mothers had fewer complications and better coping strategies [18]. Parental support is seen as helpful for a positive treatment outcome [15, 18].

Against this background, it is recommended that parents be integrated into treatment planning and decision-making [15]. Moreover, within the framework of the healthcare system, establishing networks for similarly affected girls and women is encouraged [19].

### Dealing with MRKHS coping strategies

In general, dealing with MRKHS is extremely challenging for patients. Due to the perceived threats represented by MRKHS, many patients implement potentially damaging coping strategies, such as exaggerated feminine appearance, poor choice of partners, addictive behavior, etc. [24]. Bean et al. describe coping strategies that range from complete denial of the disease to compensation through performance [15]. Moreover, some affected women run the danger of perpetually comparing themselves to other women, resulting in a negative self-assessment [24]. Dealing with MRKHS can also be influenced by so-called triggers, or negative critical experiences, that end up increasing their negative self-image [24]. One example of this is when peers within the patients’ social environment start their own families [24]. Dealing with the disease can lead to avoidance behavior and isolation, when women with MRKHS consciously avoid certain situations, such as entering a sexual relationship, and instead focus on their careers [19]. However, one review reports that career choices do not vary between women with MRKHS and healthy women [15]. Spirituality or religiousness are reported as another coping strategy to find meaning in the disease and therefore the ability to cope with it [15].

Few recommendations with regard to coping strategies have been made. As there is often a desire for interaction with other patients, self-help groups for girls and young women appear helpful [19]. In two studies, women with MRKHS were offered group therapy for psychological support [7, 16]. The main result in both studies showed that the psychological stress due to MRKHS could be significantly reduced [7, 16]. Programs offering psychological support for women with MRKHS appear to be effective.

## Discussion

This paper examines care requirements and recommendations for structuring health care during the transition from adolescence to adulthood of girls and young women with MRKHS. MRKHS is a rare disease, which is diagnosed as a malformation of the female genitals generally during adolecense, and thus during a phase in which girls and young women are also grappling with their female identities and engaging in their first sexual contacts. This clearly shows the importance of gender-sensitive psychosocial medical care during this transitional period. Although there are some clinical studies that examine somatic treatment possibilities and also increasingly incorporate aspects of sexual medicine (e.g. [34]), there are only few studies that address care requirements and give recommendations for transition management. Recommendations for the transition management of young girls and women that can lead to improvements could be distinguished in the literature. They are particularly important amid clear findings that improvements are needed in medical care and supervision within the framework of transition management. Need for improvement was found particularly for communication behavior, for information management, and for psychological support designed and tailored specifically to the target group [14, 19]. Other studies also showed that early in the diagnostic process in >40 % of the cases, false diagnoses were made, and even that incorrect treatments were initiated [6, 35]. Specific courses may be meaningful to train medical staff on how to interact with these girls and young women, as well as a transfer program specifically designed to address this rare disease and to optimize care for this target group. However the infertility is difficult for women with MRKHS to accept. In some countries there is the possibility of surrogacy as an alternative method. In Germany, it is not allowed. In recent years, new treatment options haven been developed. Brännström et al. described in the year 2014 the first clinical uterus transplantation [36]. In the year 2015 they reported about a successful livebirth after uterus transplantation [37]. Altough there exists new treatment options for patients with MRKHS, it seems to be important to keep the individual care requirements of patients with MRKHS in mind. The derived recommendations for treating MRKHS patients during the transition period should be implemented to optimize the medical care for this target group.

### Limitations

This systematic literature review has some methodical limitations. The literature review was carried out using only one database. Therefore, there is a risk that relevant studies on this subject were not considered and therefore not included in the review. A further breadth of perspective was achieved, however, as the literature search was expanded to include studies on the transition management of other diseases. Therefore, the initial focus on MRKHS may act as a “magnifying glass”, through which challenges in transition management become particularly evident, whereas simultaneously expanding the literature search was able to avoid too narrow of a perspective. This also provided for an increase in the number of studies included. However, discussion is necessary concerning the extent to which other rare diseases can be compared that have other symptoms, greater prevalence, and more experience on the part of medical staff. Another critical aspect from a methodological perspective is that, for organizational reasons, only one person (AW) carried out the full-text analysis and the critical evaluation of the studies.

## Conclusions

Altogether, the care requirements and recommendations presented here are based on relatively few studies with various study designs. It is clear that further studies are necessary in order to support the recommendations identified to improve transition management, and to systematically evaluate the effect of the interventions. This is not only true for the treatment management of MRKHS, but also in general for transition management of many other rare diseases, for which systematic evaluations are currently lacking [38].

## Abbreviations

BTP: Berliner TransitionsProgramm
MRKHS: Mayer-Rokitansky-Küster-Hauser syndrome
